# Spontaneous Arterial Thrombus and Dissection Associated With Exercise and Exogenous Testosterone Use

**DOI:** 10.7759/cureus.35936

**Published:** 2023-03-09

**Authors:** Christopher R Stewart, Cameron G Hanson, Heather A Cronovich

**Affiliations:** 1 Department of Emergency Medicine, Henry Ford Health System, Macomb, USA

**Keywords:** exercise, anabolic steroid, vascular thrombus, vascular surgery, femoral artery dissection

## Abstract

Spontaneous lower extremity arterial dissection has been linked to atherosclerotic and non-atherosclerotic causes. A 55-year-old male presented to the emergency department via emergency medical services for a chief complaint of right leg pain. He stated that he was performing leg exercises when he felt a sudden pop in his right leg followed by severe pain. His exam was remarkable for lack of ipsilateral distal popliteal or dorsalis pedis pulse by palpation or doppler. The patient was admitted to a three-year history of non-prescription testosterone injection use along with a history of prior portal vein thrombosis two years prior with anticoagulation noncompliance after one month of therapy. A computed tomography angiography of the lower extremity was performed which demonstrated complete acute occlusion of the right common iliac, and right external iliac, along with right femoral artery dissection. The patient was emergently taken to the operating room with vascular surgery where a thrombectomy with stent placement was performed. After three days in the surgical intensive care unit and nine days in the hospital, the patient was subsequently discharged from the hospital in good condition. A post-operative follow-up appointment three weeks after discharge revealed mild residual pain; however, no issues ambulating or residual weakness, and normal ankle-brachial indexes. This case highlights a unique presentation of acute limb ischemia associated with exogenous testosterone use.

## Introduction

Spontaneous lower extremity arterial dissection has been linked to atherosclerotic and non-atherosclerotic causes. Exercise has been linked to transient hypertension, combined with repetitive movements that are hypothesized to cause high turbulence and low shear stress at the sites of vessel curvature [1]. These conditions are present in the location where the external iliac artery originates from the common iliac artery and curves out of the pelvis. Atherosclerosis and vascular calcification compromise vessel integrity and have been linked to anabolic androgenic steroid abuse [2,3], including testosterone abuse which appears to be an independent risk factor for spontaneous arterial dissection. The authors detail a case of spontaneous femoral thrombus, right tibial popliteal thrombus, and right femoral artery dissection in an otherwise healthy adult male.

## Case presentation

A 55-year-old male presented to the emergency department via ambulance with a chief complaint of right leg pain. He stated the pain began after performing a leg extension exercise while at a gymnasium when he suddenly felt a “pop” in the right mid-leg. Initially, the patient denied any significant past medical history and stated his only medication was sildenafil three days ago. On examination, the patient was noted to be hypertensive with a heart rate of around 120 beats per minute. He had a notable sensory deficit from the right mid-calf distally with a lack of response to pain. The patient's leg did not visually appear pale or abnormal when compared to the left.

**Figure 1:**
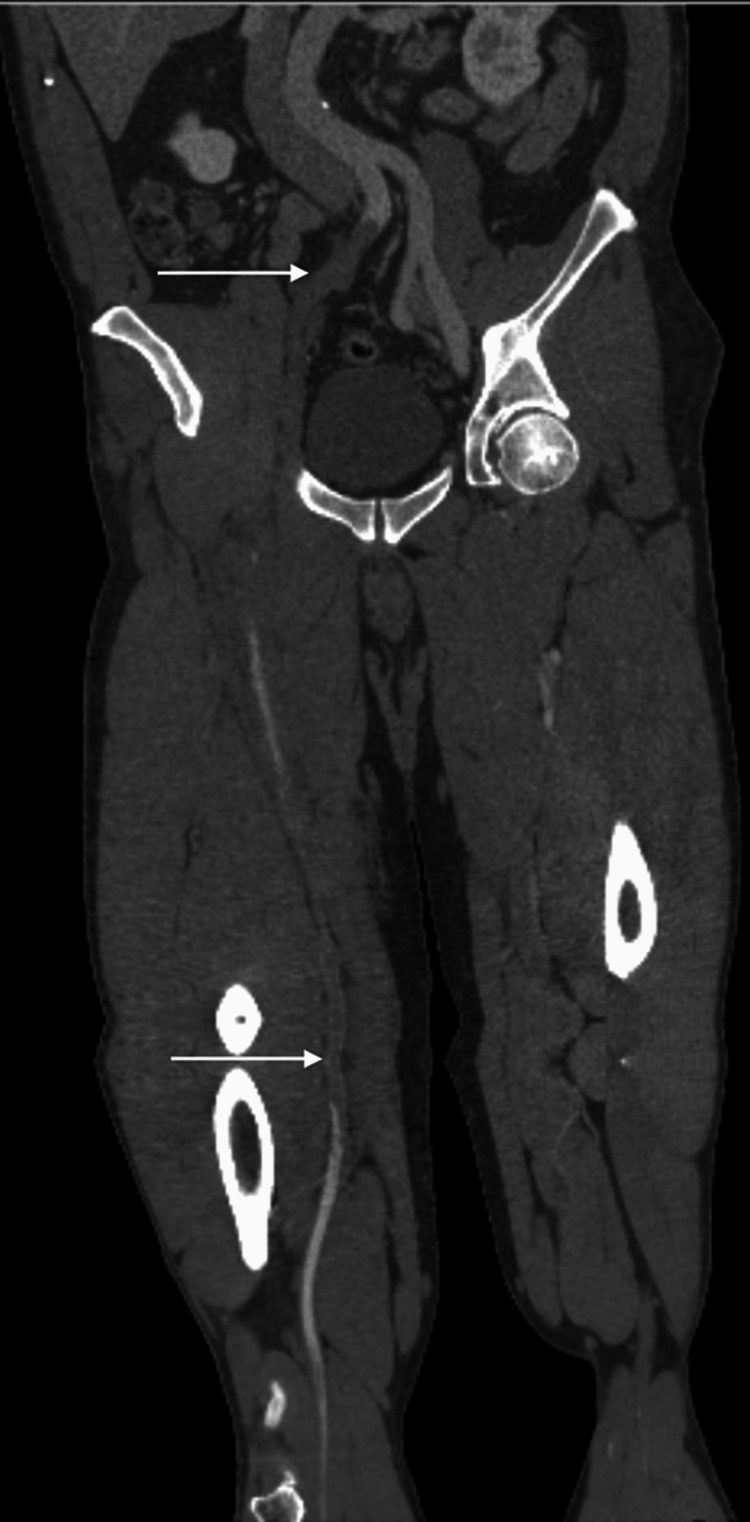
Computed tomography angiography demonstrating thrombus of the right iliac and femoral arteries

The right leg exam was also remarkable for lack of palpable posterior tibial and dorsalis pedis pulse to palpation or doppler on the ipsilateral leg. The range of motion was unrestricted. Due to a lack of distal pulses, computed tomography angiography (CTA) of the abdomen and pelvis with extremity runoff was ordered. After intravenous fentanyl administration, the patient's vitals improved with the resolution of tachycardia. The patient's CTA subsequently demonstrated complete acute occlusions of the right common iliac, and right external iliac arteries, along with findings suspicious for possible right femoral artery dissection versus acute thrombus (Figure 1).

**Figure 2:**
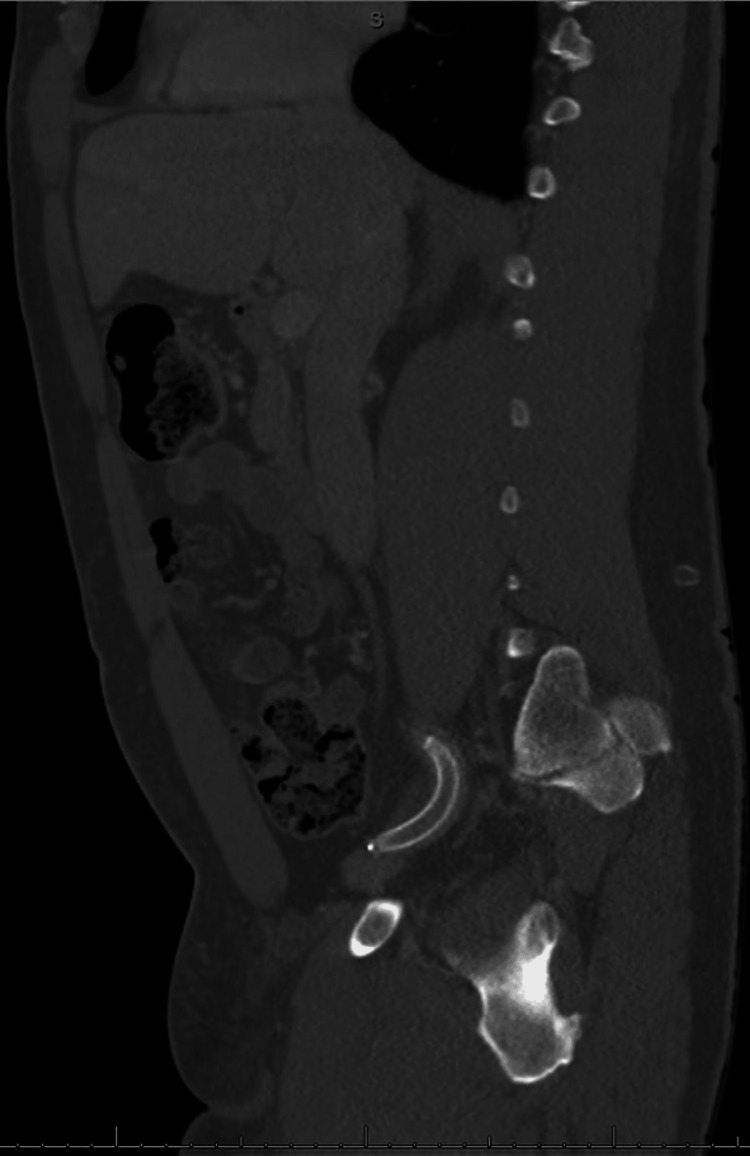
Computed tomography angiography demonstrating right external iliac stent

Patient's laboratory findings were remarkable for lactic acid of 3.4 millimole per liter (mmol/L) (normal reference: <2 mmol/L) but otherwise relatively unremarkable. At this time, vascular surgery was consulted who emergently took the patient to the operating room. Intraoperatively, the patient underwent right femoral endarterectomy with covered stent insertion of polytetrafluoroethylene interpositional graft in the right external iliac artery (Figure 2), along with exploration of the right common femoral artery with end-to-end anastomosis.

After the thrombectomy, the patient’s posterior compartment appeared tense and a subsequent fasciotomy with wound vac placement was performed. Post-operatively, the patient's exam demonstrated excellent posterior tibial triphasic flow along with monophasic dorsalis pedis Doppler flow. Surgical pathology demonstrated right femoral thrombus, right tibial and popliteal thrombus and right common femoral artery dissection. The patient was placed on 300 milligrams (mg) of clopidogrel in the post anesthesia recovery unit along with initiation of aspirin therapy. Following his procedure, the patient was transferred to the surgical intensive care unit. On postoperative day 2, the patient had worsening tension in the right posterior compartment and his fasciotomy was expanded.

 The patient had a relatively uneventful next two days in the surgical intensive care unit and was transferred to the general medical floor. The patient later revealed that for the past three years, he had been taking non-prescription intramuscular testosterone. Additionally, he noted that two years ago, he was diagnosed with a portal vein thrombosis and stopped his anticoagulation one month following this diagnosis. He remained hospitalized six additional days (nine-day total hospital stay) and was discharged in good condition on clopidogrel 75mg daily, and apixaban 5mg twice daily. Hematology workup was completed, and his polycythemia was suspected to be due to exogenous steroid use as he had no other risk factors (smoking, COPD, etc.) and no other notable causes were discovered. Three weeks following his surgery, follow-up appointment showed mild residual pain however he had no issues with ambulation and was able to tolerate physical activity and exercise without significant limitations. Ankle-brachial index performed at this time revealed right 1.0 and left 1.0. The patient was subsequently lost to follow up after this post-operative visit.

## Discussion

This case demonstrates a patient presented with multiple risk factors for arterial dissection and thrombosis including exercise-induced transient hypertension, exercise-induced repetitive strain and vascular injury, exogenous testosterone abuse, and a history of the previous clot. The etiology of the patient’s condition is likely related to these compounding and multifactorial risk factors. Hypertension has long been linked to vascular injury, and exercise (especially exercise with heavy weights) leads to transient hypertension during the time of the event. Such pressures in an area of natural turbulence, such as the branches and curves in the region of the common iliac, have been thought to contribute to arterial dissection [1,4]. Atherosclerosis, however, remains a strong risk factor for vascular disease and the abuse of androgenic anabolic steroids (such as testosterone) has been shown to increase that risk [3-5]. Previous studies have noted two different mechanisms for this contribution, namely steroid-induced lipid metabolism disorder (leading to decreased HDL and increased LDL) and elevated homocysteine levels [3]. It has also been noted that androgenic anabolic steroids can increase the degree of vascular calcification, ultimately reducing tissue elasticity. These changes can lead to increased tissue damage due to decreased elasticity and to rupture of atherosclerotic plaques due to the severe transient hypertension found in heavy weightlifting. [3,6]. Additionally, the patient’s pathological specimens were also notable for multiple thrombi. With regards to the patient’s past medical history, he had demonstrated prior risk factors for the development of thrombus, including a history of previous portal vein thrombosis without adherence to anticoagulation, and a three-year use of androgenic anabolic steroids. These substances directly affect coagulation and fibrinolysis through enhanced platelet generation and aggregation and regulation of Thromboxane A2 (TXA2) receptor density [3]. Spontaneous lower extremity arterial dissections following exercise are rare, with relatively few cases available in the literature [1,4-6]. While some of the case studies illustrate an individual with non-atherosclerotic processes, the individual in this case presents with both atherosclerotic and non-atherosclerotic risk factors. These numerous risk factors likely compounded to create his presentation.

## Conclusions

Spontaneous lower extremity dissection and acute thrombus formation is a rare condition associated with multiple etiologies. Among the rarest, non-atherosclerotic causes may be missed in patients without a history of coronary artery disease or similar risk factors. This case highlights a unique presentation of acute limb ischemia associated with exogenous steroid use. It is important for emergency physicians to be aware of this potential complication and to educate patients on the risks of exogenous steroid use.
